# Interfraction Variation and Dosimetric Changes in Patients With Cervical Cancer Treated With Intracavitary Brachytherapy

**DOI:** 10.1200/JGO.2016.008557

**Published:** 2017-07-06

**Authors:** Jayson L. Co, Maureen R. Bojador, Michael Benedict A. Mejia, Teresa T. Sy Ortin, Domingo E. Ganzon

**Affiliations:** All authors: University of Santo Tomas Hospital, Manila, Philippines

## Abstract

**Purpose:**

Intracavitary brachytherapy is integral in the treatment of cervical cancer. Because of interfraction variation, the current standard is replanning with every fraction. This study aimed to determine whether there was a difference in relative dosimetry if the source position and dwell time of the first fraction were applied to subsequent fractions.

**Materials and Methods:**

The authors performed a retrospective review of charts and films from 2007 to 2012. Eligible cases were patients with cervical cancer treated with brachytherapy with the same dose prescription to point A. Replanning was done on the first set of orthogonal plates. Source position and dwell time were subsequently applied to the remaining fractions using actual films.

**Results:**

Twenty-nine patients were included in this study. The results showed that cervical, rectal, and bladder dose between the actual plan and the hypothetical plan were not statistically different. In the hypothetical plan, the source activity and dwell time of the first plan were applied to the orthogonal films of the subsequent fractions and showed no significant difference in all dose points.

**Conclusion:**

The results of this study showed proof of concept of the safety of using the source position and dwell time of the first plan for subsequent fractions. Until further studies are performed (also using three-dimensional planning software), the concept should be considered investigational because of the small sample size of the study. Until such research is performed, it is still strongly recommended that replanning be performed with every fraction whenever it is feasible.

## INTRODUCTION

Despite effective screening and treatment of preinvasive lesions, cervical cancer remains the third most common gynecologic malignancy worldwide and the second most common in the Philippines.^1,2^

With the advent of remote afterloading devices, high dose–rate brachytherapy (HDRBT) has become the standard of care in treatment of gynecologic malignancies. HDRBT allows for shorter treatment times per fraction as well as delivery in ambulatory settings, and is thus associated with greater patient convenience and comfort and lesser risk for venous thromboembolism. The relative doses to the tumor volume and organs at risk depend on patient anatomy, the type of placement, and stability of the applicator. Recent years have seen the introduction, advancement, and proliferation of computed tomography (CT)-based, image-guided techniques. Although these techniques have theoretical dosimetric advantages, their impact on improving tumor control and decreasing late toxicities has yet to be definitively demonstrated. In addition, cervical malignancies are more prevalent in developing economies, where access to more advanced technologies is severely limited. The longer procedure time also limits patient throughput in these resource-limited settings.

Significant interfraction variations may result from dramatic tumor regression or progression during brachytherapy. Thus, replanning with every fraction has been proposed and is currently recommended by the American Brachytherapy Society (ABS).^3,4,5,6^ A retrospective study showed that significant variation in applicator positions during fractions led to different doses delivered to the bladder and rectum, but with no significant difference in radioequivalent doses. Furthermore, tumor size did not correlate with the magnitude of discrepancy among applicator positions and rectal and bladder points.^7^ The possibility of eliminating replanning in certain subsets of patients may reduce both waiting and sedation times for patients as well as medical staff time. These evidences were based on orthogonal-based brachytherapy and may not be directly applicable to three-dimensional brachytherapy and image-guided brachytherapy. This is still relevant because despite the increasing adoption of three-dimensional brachytherapy and image-guided brachytherapy in high-income countries, a number of centers still use orthogonal systems, especially in low- to middle-income countries.

To our knowledge, no local study has examined the correlation among interfraction variation in applicator placement, tumor volume regression, and its relationship with dosimetry. The investigators sought to determine whether there would be significant change in relative dosimetry if the source position and calculated dwell times generated during the first plan were applied in subsequent fractions for patients treated previously in our hospital with intracavitary brachytherapy for cervical cancer.

## MATERIALS AND METHODS

This is a retrospective review of two-dimensional brachytherapy plans for patients with cervical cancer treated at our cancer institute between 2007 and 2012. The protocol adheres to good clinical practice and the Declaration of Helsinki. Institutional review board approval was obtained before commencement of this study. Eligible cases were patients with histologically confirmed, nonmetastatic cervical carcinoma treated with definitive concurrent chemoradiation with at least four fractions of brachytherapy using Henschke applicators with the same dose prescription to point A for all fractions. Uterine dilatation length should not differ by > 1 cm among brachytherapy fractions. Excluded were patients with previous pelvic surgery, interfraction difference in uterine dilatation length > 1 cm, different point A dose prescriptions among fractions, and use of different tandem curvatures among fractions.

### Brachytherapy Procedure

HDRBT was given either after the completion of whole pelvic external beam radiotherapy (EBRT) with 50 Gy or after 40 Gy if the cervical mass had decreased satisfactorily, according to the discretion of the attending gynecologic oncologist and radiation oncologist.

The brachytherapy procedure was in accordance with the guideline by the ABS.^8^ All patients were required to receive intravenous general anesthesia with ≥ 8 hours of fasting before the procedure. Before each brachytherapy, clinical examination and measurement of the tumor were done while the patient was under anesthesia. The Henschke applicator was used, which is composed of one intrauterine tandem and two ovoids. The degree of tandem curvature used was at the discretion of the oncologist; however, to be included in the study, the same curvature should have been used throughout all of the fractions. A urinary catheter was placed in the bladder and instilled with 7 cm^3^ of radiopaque solution, and a rectal tube with wire marker was applied to the rectum. Vaginal packings were applied to move the bladder and the rectum away from the applicator.

Orthogonal films using anteroposterior and lateral views were taken after insertion of the applicator. Digitization and dosimetric planning were performed by the same physicist, using the Nucletron microSelectron V2 brachytherapy planning software. The plans were reviewed and approved. The prescription, optimization, and monitoring points were designated according to the International Commission on Radiation Units & Measurements #38 guidelines, except for the rectal points, which were defined as two points 1 cm apart along a radiopaque rectal wire marker, that were closest to the applicator. Ideally, the rectal point is marked by 0.5 cm posterior to the vagina wall instilled with radiopaque solution, because use of intrarectal wire may underestimate the dose delivered to the rectum.^9,10^ During the establishment of brachytherapy in our institution, the radiopaque solution used for brachytherapy was not readily available; thus rectal wire was used. The cervical point dose is defined as 1 cm superior and lateral to the cervical os. The prescribed dose was 6.5 Gy delivered at point A in all patients. The plans were optimized such that the bladder dose was < 6 Gy and the rectal dose was < 5 Gy per fraction. The actual brachytherapy was given using the iridium-192 Nucletron afterloader machine.

Using the previous orthogonal films, a hypothetical replanning was done for the purpose of this study. Upon approval of the hypothetical first-fraction plan, the source position and calculated dwell duration were applied to the brachytherapy applicator positions using the orthogonal films of the second, third, and fourth fractions. The dose delivered to point A, point B, the bladder, the rectum, and the cervix were then determined.

### Statistical Analysis

Demographic data of participants were summarized using descriptive statistics (the mean value and standard deviation). Normality of each distribution was determined using the Shapiro-Wilk test. Analyses of differences between groups were done using the χ^2^ test for independence, Wilcoxon signed rank test, or Friedman test. *P* values ≤ .05 were considered significant.

## RESULTS

Between January 2007 and December 2012, a total of 153 patients with cervical cancer were treated with definitive radiotherapy consisting of whole pelvic EBRT and brachytherapy with or without concurrent chemotherapy. Upon review of the charts, 124 patients were excluded as the result of differences in depth of uterine tandem insertion (n = 55); differences in localization of point A (n = 12); differences in uterine tandem angulation among the four fractions of brachytherapy (n = 5); and either missing, less than four fractions of brachytherapy, or incomprehensible or faded radiographs (n = 52). Twenty-nine patients (116 brachytherapy applications and films) were eligible for the study.

The patients’ characteristics and initial tumor size did not differ significantly among age, histology, and stage (Table 1). It was noted that throughout the EBRT and brachytherapy, there was a significant decrease in the clinical tumor size (Table 2).

**Table 1:**
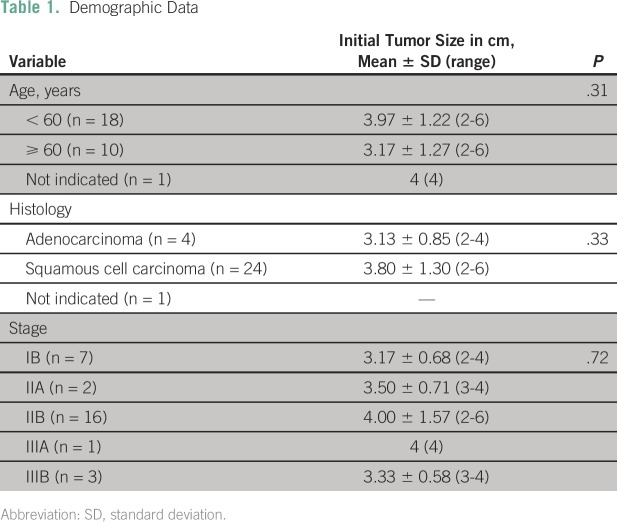
Demographic Data

**Table 2:**
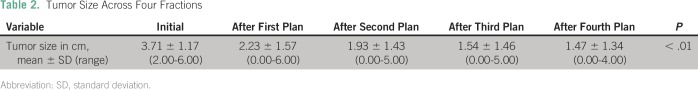
Tumor Size Across Four Fractions

Comparison of the actual plan and the hypothetical plan was done (Table 3). The result showed that the cervix doses for the actual plan were 15.32 ± 2.63 Gy, 15.46 ± 2.52 Gy, 15.14 ± 2.99 Gy, and 14.94 ± 1.71 Gy and for the hypothetical plan were 14.68 ± 3.56 Gy, 14.62 ± 5.90 Gy, 12.82 ± 5.06 Gy, and 14.22 ± 3.33 Gy for the first, second, third, and fourth fractions, respectively. There was no significant difference in the cervix dose between the actual plan and the hypothetical plan (*P* > .05). The bladder doses for the actual plan were 3.37 ± 1.26 Gy, 3.29 ± 0.98 Gy, 3.54 ± 1.16 Gy, and 3.29 ± 1.51 Gy and for the hypothetical plan were 3.40 ± 1.56 Gy, 3.55 ± 1.23 Gy, 3.57 ± 1.26 Gy, and 3.33 ± 1.34 Gy for the first, second, third, and fourth fractions, respectively. Again, there was no significant difference in the bladder dose between the actual plan and the hypothetical plan (*P* > .05). The rectal doses for the actual plan were 2.99 ± 0.85 Gy, 2.82 ± 0.78 Gy, 2.91 ± 0.93 Gy, and 2.94 ± 1.00 Gy and for the hypothetical plan were 2.75 ± 0.81 Gy, 2.95 ± 1.01 Gy, 2.87 ± 0.97 Gy, and 3.05 ± 1.17 Gy for the first, second, third, and fourth fractions, respectively. There was again no significant difference in the rectal dose between the actual plan and the hypothetical plan (*P* > .05; Table 3).

**Table 3:**
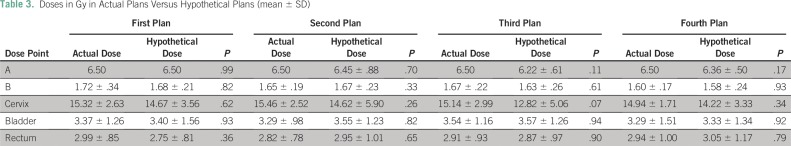
Doses in Gy in Actual Plans Versus Hypothetical Plans (mean ± SD)

We determined the doses delivered to the organs at risk relative to that delivered to point A in the hypothetical plan. The bladder dose/point A dose ratio and the rectal dose/point A dose ratio exceeded 80% in six and three plans, respectively.

In the hypothetical plan, the source activity and dwell time of the first plan were applied to the orthogonal films of the second, third, and fourth brachytherapy applications (Table 4). The doses delivered to point A were 6.45 ± 0.88 Gy, 6.21 ± 0.61 Gy, and 6.36 ± 0.50 Gy for the second, third, and fourth fractions, respectively. In each case, the dose was not significantly different from that obtained in the first fraction (*P* > .05).

**Table 4:**
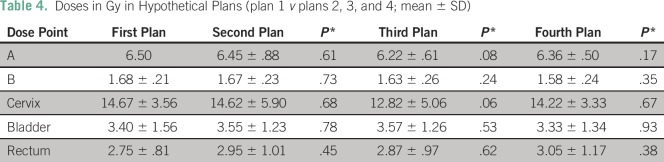
Doses in Gy in Hypothetical Plans (plan 1 *v* plans 2, 3, and 4; mean ± SD)

In the hypothetical plan, the doses delivered to the cervix were 14.62 ± 5.90 Gy, 12.82 ± 5.06 Gy, and 14.22 ± 3.33 Gy for the second, third, and fourth fractions, respectively. In each case, the dose was not significantly different from that obtained in the first fraction (*P* > .05). The doses delivered to the bladder were 3.55 ± 1.23 Gy, 3.57 ± 1.26 Gy, and 3.33 ± 1.34 Gy for the second, third, and fourth fractions, respectively. Each fraction was compared with the plan of the first fraction; again, there was no significant difference in dose delivered to the bladder point (*P* > .05). The dose to the rectum was 2.95 ± 1.01 Gy, 2.87 ± 0.97 Gy, and 3.05 ± 1.17 Gy for the second, third, and fourth fractions, respectively. Each fraction was compared with the plan of the first fraction; there was no significant difference in dose delivered to the rectum point (*P* > .05).

## DISCUSSION

The results of this study showed that there was no significant difference between the actual (implemented) plan and the hypothetical plan with regard to the point dose to the cervix, bladder, and rectum. Moreover, when the source position and calculated dwell time of the hypothetical plan for the first fraction were applied to the application of the second, third, and fourth plans, there was no significant difference between the point doses. A previous study showed that the bladder and the rectal points differed among fractions in relation to bony landmarks but, despite this, the total radioequivalent dose delivered to the rectum and bladder remained the same.^7^

The current recommendation of the ABS is still to replan with every fraction.^8^ Care should be taken when planning to use orthogonal films because these use point doses rather than volumes; as such, the maximum dose may not be estimated. There is a possibility of overdosing the rectal dose up to two to three times what is reported on isodose curves. Another advantage of replanning is the ability for dose optimization. Compared with optimized plans, nonoptimized plans may increase overdoses at the vaginal surface, bladder, and rectum.^11^ The results of this study do not recommend the elimination of repeat planning when it is feasible. This analysis, however, highlights that brachytherapy can potentially be given safely if similar conditions are achieved between fractions. This should not be misconstrued as recommending that the parameters during the first insertion be replicated, when this can potentially be improved during subsequent fractions (eg, greater uterine tandem insertion depth); however, the findings may be useful for centers with limited resources and facilities.

The tumor size may regress during the course of EBRT and brachytherapy. Bahena et al^12^ reported that tumor regression may be attributed to a difference of only one standard deviation between positions of the applicator. There are conflicting data with regard to tumor regression and dose to the bladder.^13,14^ In vaginal-cuff brachytherapy, vaginal fibrosis, rather than presence of tumor, contributes to change in dosimetry.^15^ Various efforts have been made to decrease the dose to the rectum and bladder, including the use of balloon catheter and lithotomy positioning.^3,16^ The study only included patients with uterine dilatation ≤ 1 cm, as defined by the level of the flange. The reason for limiting the population was to minimize possible anatomic variation among fractions. Additional studies may be conducted to investigate the effect of different magnitudes of uterine dilatation on dosimetry if replanning is not done.

In most centers, variations in placement techniques exist among practitioners of brachytherapy (ie, radiation and gynecologic oncologists). Operator-dependent differences in applicator insertions and vaginal packing also contribute to displacement of the applicator.^4^ Although the same surgeon packed the vaginal vault with gauze, the retrospective nature of our study fails to control the method and the degree of vaginal packing. Another factor is the type of applicators. Some cases may require use of a different applicator or a different tandem curvature applicator among fractions to best suit the anatomy of a given patient. This is one of the scenarios wherein the results of this study may not apply.

A current trend in gynecologic brachytherapy is the use of image-guided brachytherapy. A 2014 survey by the ABS showed that in the United States, 95% and 34% of the respondents used CT scans and magnetic resonance images for guidance, respectively. Despite this, 46% still prescribed to point A rather than volume.^17^ However, most cases of cervical cancer are in less developed and developing countries, where resources are limited. Using the International Atomic Energy Agency’s Directory of Radiotherapy Centres and GLOBOCAN data, a report was able to show that 91% of the need for HDRBT in low- to middle-income countries is covered with present equipment. But when this was stratified in the Asia Pacific and African regions, only 38% to 45% of HDRBT needed is addressed.^5^ In fact, the International Federation of Gynecology and Obstetrics has adopted clinical staging with limited diagnostics, taking into consideration that modern facilities may not be available in these low-resource countries.^6^ Our findings may still be applicable to those centers that use orthogonal planning systems for intracavitary brachytherapy.

There are limitations in this study that should be noted. Only 19% of patients were eligible from the retrieved charts; 29% were eligible when correction for the incomprehensible radiographs was taken into consideration. This stems from the strict inclusion criteria and inherent uncontrolled design of the study. We recommend a larger sample size to exclude the possibility of accepting the null hypothesis as the result of an underpowered study. In the cervical dose, there seemed to be a trend toward a significant difference, and this may be further investigated with a larger sample. The anatomic variation as the result of tumor regression and some differences in tandem and ovoid insertion may be the reasons for the trend. The relatively small sample size and retrospective nature of the analysis limit the applicability of the findings as part of standard treatment. Another limitation is the use of rectal wire marker for the rectal point, which may not be standard in some institutions. Because this is a retrospective study, we were not able to control this variable. We recommend that the standard International Commission on Radiation Units & Measurements definition be used in further studies. The results demonstrated proof of concept that, with certain parameters and logistics, replanning may be waived; however, the authors do not recommend it as a standard of care. Similar dosimetric studies using larger samples are warranted. It would also be of interest to perform similar analyses on systems using CT-based software.

In conclusion, the results of this study showed proof of concept of the safety of using the source position and dwell time of the first plan for subsequent fractions. Until additional studies are performed (also using three-dimensional planning software), the concept should be considered investigational because of the small sample size of the study. Until such research is performed, it is still strongly recommended that replanning be performed with every fraction whenever it is feasible.
